# Policy Reform on the Qualification Pathway of Internationally Educated Nurses in Greenland and Its Relationship With the Danish System: A Qualitative Discourse Analysis

**DOI:** 10.1177/15271544241245975

**Published:** 2024-04-24

**Authors:** Floro Cubelo

**Affiliations:** 1 School of Wellbeing and Culture, Oulu University of Applied Sciences, Oulu, Finland; 2 International Coordination & Management Affairs, The Filipino Nurses Association in the Nordic Region, Oulu, Finland; 3 Department of Nursing Science, Faculty of Health Sciences, University of Eastern Finland, Kuopio, Finland

**Keywords:** foreign nurses, government, internationally educated nurses, nurses, nursing management, policy

## Abstract

The nursing profession in Greenland, particularly in rural and remote areas, faces challenges due to geographical limitation and a lack of interdisciplinary collaboration. The registration process and status of internationally educated nurses (IENs) in Greenland are unclear. This article aimed to analyze existing policies and propose recommendations for an independent registration process for IENs in Greenland. A qualitative discourse analysis was used to critically discuss existing policies and regulations governing nursing registration in Greenland. Relevant legislation, government reports, and official documents were reviewed. Legislative regulations protect the title of registered nurse in both Greenland and Denmark. To work in Greenland, an IEN must have a residence permit. With recent health agreements between Greenland and Denmark, both countries have streamlined the permit acquisition process for foreign healthcare professionals, making it more accessible. However, the process of acquiring a license to work as a registered nurse for IENs lacks clarity. Policy reform is needed to establish a group of diverse nurse experts under the National Board of Health responsible for the assessment and registration of IEN qualifications. There is also a need for a bridging education program or national licensure examination which could facilitate faster IEN recognition. Mutual recognition of nurse licenses between Greenland and Denmark should be established to ensure efficient healthcare delivery and maintain professional standards. Embracing IENs can address nursing shortages and improve healthcare services in Greenland.

## Background

Historical records suggest human arrival in Greenland around 2500 BC, followed by successive waves of migration from North America. Inuit migration from Asia in the 13th century marks a significant lineage that endures today, constituting 88% of the population, predominantly Kalaallit or Danish-Inuit mixed ancestry. The remaining 12% primarily comprises individuals of European descent, predominantly Danish (Huang, 2023).

Greenland, although geographically located in North America, maintains autonomy as a constituent part of the Kingdom of Denmark. Over about a thousand years, it has established political and cultural ties with Europe. Initially a Danish colony from 1721, Greenland became fully integrated into Denmark in 1953. The progression toward self-governance began in 1979 with Home Rule, followed by expanded Self-Rule in 2009, signifying a transfer of decision-making authority and responsibilities to the Greenlandic government. This transitional framework allows Greenland to progressively assume additional responsibilities from Denmark as it becomes ready (Huang, 2023).

Despite following the Danish system, the nursing profession in Greenland, especially in rural and remote areas, faces many challenges. In rural and remote areas of the country, nurses collaborate with various professionals through telemedicine, email, and telephone due to geographical limitations (Hounsgaard et al., 2013; Hounsgaard & Seibæk, 2018). However, there is a lack of interdisciplinary collaboration, and issues arise from a monodisciplinary approach, with each nurse operating within their learning and training (Hounsgaard et al., 2013). As of 2013, nursing stations typically had one nurse and a health assistant, while healthcare units have a larger team of nurses for knowledge sharing and collaboration (Hounsgaard et al., 2013). However, these nursing stations are no longer operational. Periodically, a single nurse may be present in a settlement for a brief duration (H. Hansen, personal communication, March 9, 2024).

In terms of the healthcare system, Dronning Ingrids Hospital in Nuuk serves as Greenland's central hospital, while specialized care is referred to Rigshospitalet in Copenhagen, Denmark. Patient transport logistics in Greenland are complex, often taking days to reach appropriate-sized hospitals. Air Greenland facilitates domestic patient transfers, with Dronning Ingrids Hospital healthcare personnel managing critical flights, and patients typically transported to Copenhagen via Kangerlussuaq Airport by aircraft for advanced care (Gunnarsson et al., 2015).

This complex healthcare infrastructure plays a crucial role in supporting Greenland's nursing education system. According to Møller (2016), nursing education in Greenland follows the European Credit Transfer and Accumulation System (ECTS), which comprises 150 ECTS. When Greenland took over the healthcare system of the country from Denmark, it also established its 4-year nursing education equivalent to a Bachelor of Science in Nursing. The program is financed by the Greenland Self-Rule government and admitted the first batch of nursing students in 1994 where the curriculum is inspired by the Danish nursing program (Møller, 2016).

In 2015, the nursing workforce consisted of around 325 individuals, including 140 who received their education in Greenland. Before the initiation of the nursing education program, approximately 30 native Greenlandic nurses were trained in Denmark (Møller, 2016). At the beginning of 2024, the nursing registry, as per the National Board of Health, documented 5,795 nurses, with 205 having undergone education within Greenland (H. Hansen, personal communication, March 04, 2024). Among these figures, the total number of nurses employed at any given time does not exceed a maximum of 325, and this count has remained unchanged since 2015 (H. Hansen, personal communication, March 9, 2024).

In 2022, 2,040 foreign nationals resided in Greenland, constituting 3.5% of the total population. Key immigrant groups include individuals from the Philippines, Thailand, and Iceland. Conversely, the number of Greenland-born individuals residing in Denmark increased from 14,908 in 2012 to 16,801 by 2022 (Kleemann, 2023). In the nursing sector, 74 individuals received their basic nursing education outside of a Nordic country (H. Hansen, personal communication, March 09, 2024). Nurses educated in Greenland intending to practice in Denmark require Danish authorization from the Danish Patient Safety Authority, while those intending to practice solely in Greenland are exempt from registration in Denmark (National Board of Health, 1995).

**Figure 1.**
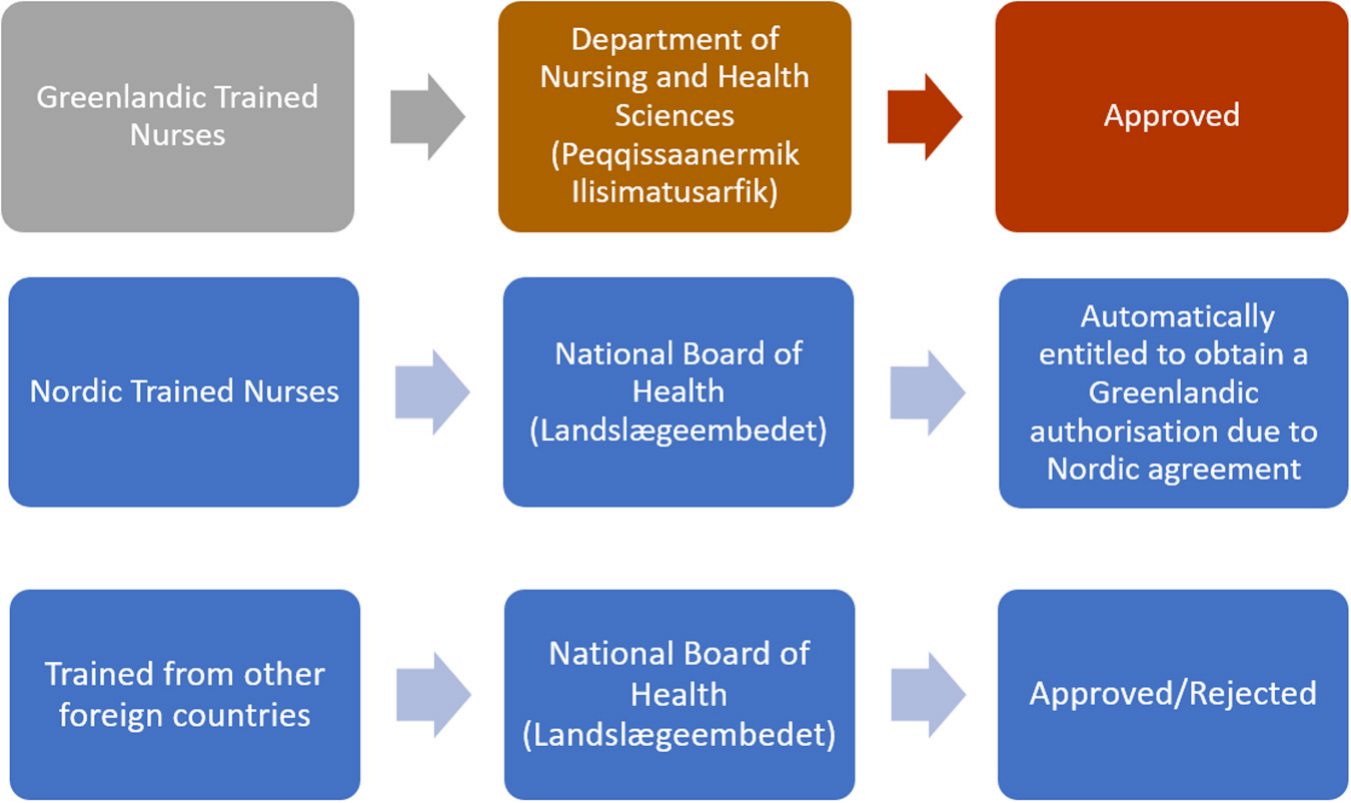
Application process for nurses trained in Greenland, the Nordic region, and other foreign countries.

The pathway for internationally educated nurses (IENs) educated outside the Nordic countries to become registered nurses (RN) in Greenland has not been extensively studied. Due to Greenland's historical ties with Denmark (Kočí & Baar, 2021), Greenlandic authorities use the Danish requirements as guidelines when issuing authorizations to IENs educated outside the Nordic region (H. Hansen, personal communication, March 10, 2024). However, recognizing Greenland's independence necessitates strengthening its own registration system. The National Board of Health (1995) handles the authorization process, requiring an individual assessment for applicants educated outside Greenland and the Nordic countries (see Figure 1). The assessment considers educational background, study duration, curriculum vitae, and confirmation from legal authorities within the last 3 months. Notably, there is no explicit mention of Greenlandic language certification (National Board of Health, 1995), despite Greenlandic being the official language (Kleemann, 2023), and the confirmation process for IENs’ skills and competencies remains unspecified (National Board of Health, 1995).

Given Greenland's growing older adult population (Albertsen et al., 2021), a shortage of healthcare personnel (Exner-Pirot et al., 2018; Pedersen et al., 2022), recruitment challenges (Møller, 2016), and the persistent public health challenges faced by Inuits, including adverse childhood experiences, addictive behaviors such as alcohol misuse, mental health issues leading to suicide, dietary concerns and obesity, and smoking (Bjerregaard & Larsen, 2018), there is a compelling need to address these multifaceted challenges. In a recent study, nurses from foreign backgrounds in the Nordic region appeared to be crucial members of the nursing workforce during the COVID-19 pandemic. Despite facing physical and mental challenges, they served as front-line workers, especially during the initial wave of the pandemic when resources were scarce. Their skills played a vital role in addressing the nursing shortage throughout the region (Cubelo et al., 2024). In response to these considerations, there is a need to reform and enhance the pathway for IENs.

With its historical independence from Denmark, Greenland is confronted with the critical need to assert its own national identity while simultaneously enhancing collaboration with Denmark. This collaboration is particularly crucial in establishing more transparent pathways for IENs, especially those educated outside the Nordic region. A notable challenge lies in defining how IENs can practice as RNs in Greenland and ensuring the alignment of their skills and competencies with the country's legislative framework.

## Aim

This article aims to discuss the existing policies and propose recommendations for a policy reform that ensures an independent and clear registration process and credentialing pathway for IENs in Greenland.

## Method

A qualitative discourse analysis was used to discuss the process of qualifying as an RN in Greenland. This was achieved by establishing connections between existing policies that protect the professional title within the country and their implications for public health. The discursive approach was an appropriate method in this article to understand how actors construct and modify public policies and how they affect the social norm (Durnova & Zittoun, 2013). Initially, the author engaged with national health authorities and managers experienced in matters related to IENs. Subsequently, the author sought guidance from various official government websites on the qualification pathway for IENs in Greenland. Additional verification and discussions were conducted through phone calls. Following this, data retrieval focused on examining pertinent legislation, government publications, and other relevant documents to understand the political relationship between Denmark and Greenland. Then, further discussion focused on the current policies and regulations governing the registration and certification procedures for nursing in Denmark and Greenland and the IEN authorization process.

## Results

Official documents were retrieved from the official government websites of Denmark and Greenland. Five official legislative documents were retrieved, supported by press releases of existing updates in legislation and policies to ensure that the information is up-to-date (see Table 1). Greenland's self-governance is established through the Act on Greenland Self-Government, which grants legislative, executive, and judicial powers to the Government of Greenland, the Parliament of Greenland, and the Self-Government authorities’ courts (International Labour Organization, 2009; Kleemann, 2023; The Prime Minister's Office, n.d.). Legislative regulations protect the RN title (Greenland Self-Government, 1995; National Board of Health, 1995) and employment in both Greenland and Denmark. Employers seeking foreign employees in Greenland must obtain permission from the local municipality (Kleemann, 2023). However, a recent Health Agreement has facilitated easier access to work and residence permits for foreign health personnel, reducing processing time and eliminating the need for approval from the Government of Greenland (SIRI, 2023).

**Table 1.** Selected Relevant Legislations and Regulatory Bodies, Their Applicability, Limitations, and How They Relate to IENs in Greenland and Denmark.

Regulation/regulatory giving bodies	Limitations and specifics	Relevance to internationally educated nurses
GREENLAND		
Parliamentary Regulation No. 7 Pursuant to § 1 and § 3 of Act no. 369 of 6 June 1991 on the health service in Greenland	Authorization granted to those who completed the Greenlandic nursing examination Denial possible based on physical or mental deficiencies or gross incompetence Refusal under specific circumstances (Danish Criminal Code) Overseen by the Government of Greenland	Governs the authorization and activities of nurses in Greenland. IENs completing Greenlandic nursing examination eligible for authorization
Greenland Parliament Act no. 27	Priority for Greenlandic workers in employment Employers apply for permission to hire foreign workers Utilization of foreign labor allowed in scarcity of qualified domestic workers	Influences employment opportunities in Greenland. IENs may be considered based on the shortage of locally qualified workers
National Board of Health	Regulatory body for healthcare professionals	IEN qualification assessment and registration
DENMARK		
Danish Patient Safety Authority Danish Authorization Act	Regulates and grants licenses to health professionals License required for nurses educated outside EU/EEA	Governs licensure for nurses in Denmark, including those educated outside the EU/EEA and intending to practice in Greenland
Executive Order No. 731 of 8 July 2019 on authorization of healthcare professionals and healthcare practice	Authorization granted to those who completed Danish nursing examination or equivalent foreign examination The Ministry for Interior and Health defines nursing activities and boundaries	Establishes regulations for nursing practice in Denmark, including authorization criteria and Ministerial authority over nursing activities Relevant for IENs educated outside Denmark
Health Agreement	Facilitates easier access to work and residence permits for foreign health personnel Permits granted without approval from the Government of Greenland under certain conditions	Expedites permit issuance for healthcare professionals, including IENs, making it easier to work in Greenland
	Expedited work permits for healthcare professionals Eligibility tied to collective bargaining agreement	Impacts IENs seeking employment in positions requiring Danish medical authorization Full-time employment under collective bargaining agreement necessary

*Note*. EEA=European Economic Area; EU=European Union; IEN=internationally educated nurse.

## Greenland's Self-Governance

According to the Act on Greenland Self-Government no. 473 of June 12, 2009, it is acknowledged that the people of Greenland possess the right of self-determination under international law. The purpose of this Act is to promote equality and mutual respect in the partnership between Denmark and Greenland, with the agreement based on an equal partnership between the Government of Greenland and the Danish Government. Under Chapter 1 of the Act, the Greenland Self-Government authorities are empowered to exercise legislative and executive power in the areas of responsibility they have assumed. The courts established by the Self-Government authorities have jurisdiction over judicial matters in Greenland across all fields of responsibility. Thus, legislative power rests with the Parliament of Greenland, executive power lies with the Government of Greenland, and judicial power is vested in the courts of law (International Labour Organization, 2009).

## Legislations Protecting the Registered Nurse Title and Employment

### In Greenland

Parliamentary Regulation No. 7, dated October 30, 1995, addresses the authorization and activities of nurses in Greenland. This regulation, which amends and replaces previous information, outlines the right to practice as a nurse and the authorization requirements. According to Section 1, only individuals who have obtained authorization following Section 2 or Section 3 are permitted to practice as nurses and use the title of nurse. Section 2 specifies that individuals who have successfully completed the Greenlandic nursing examination are eligible for authorization as nurses on application. However, authorization may be denied if an individual is considered dangerous to others due to physical or mental deficiencies or gross incompetence. Additionally, an authorization may be refused under circumstances outlined in Section 114, subsection 2 of the Greenlandic Criminal Code. The granting of authorization is overseen by the National Board of Health under the Government of Greenland (Greenland Self-Government, 1995).

However, under Administrative Regulation, no. 15 of April 22, 1998 concerning section 3(4) of Parliamentary Regulation no. 7 of October 30, 1995 on the authorization and activities of nurses, foreign nurses seeking employment in the Greenlandic healthcare system undergo an assessment by the employing authority, considering personal, professional, and other qualifications, including at least 1 year of practical and linguistic experience as a nurse from a Nordic country, language skills equivalent to Danish (Danskprøve 2), or proficiency in Greenlandic or a closely related dialect (Inuktitut) (Greenland Self-Government, 1998).

### In Denmark

The Danish Patient Safety and Authority (STPS) regulates and grants licenses to health professionals who seek to work in the country. According to the Danish Authorization Act, nurses educated outside the European Union (EU)/European Economic Area (EEA) must apply for a license before practicing the nursing profession (STPS, n.d.).

Following section 2(4) of the Act on Nurses (Consolidating Act no. 66 of February 27, 1979, as amended by Section 35 of Act no. 217 of April 23, 1986), as stated in the Executive Order on the Nursing Program, the following regulations are established. Chapter 1 focuses on the purpose and structure of the program, aiming at allowing students to develop the personal and professional qualifications necessary for nursing practice. Additionally, the program seeks to provide prerequisites for effective collaboration, professional growth aligned with scientific and social advancements, and the advancement of the nursing profession. The nursing program, conducted at educational institutions with an approved education scheme as specified in Section 10, has a duration of 45 months, comprising a combination of theoretical and practical training in a ratio of 5:4 (Ministry of Interior and Health, 1990).

In Chapter 13 of the legislation governing nursing, specifically Section 54 of Executive Order No. 731 of July 08, 2019, authorization as a nurse is granted to individuals who have successfully completed the Danish nursing examination or a foreign examination deemed equivalent, as outlined in Sections 2 and 3. It is important to note that only those who have obtained authorization as nurses have the right to use the title of the nurse. Furthermore, the Minister for Interior and Health of Denmark has the authority to establish regulations regarding the practice of nursing activities and define their limits in Denmark (Ministry of Interior and Health, 2019).

## Limitations with the Danish Legislation

In Executive Order No. 731, under Title VI, specifically in Chapter 28 of the legislation, it is stated that the Act, referring to the specific legislation being discussed, does not apply to Greenland and the Faroe Islands. However, through a Royal Decree, the Act can be enforced in its entirety or partially, for the Faroe Islands, with the necessary deviations to accommodate their unique conditions (Ministry of Interior and Health, 2019).

This statement implies that the Act being referred to does not directly apply to Greenland and the Faroe Islands. However, there is a provision that allows for the Act to be implemented in the Faroe Islands, either in its entirety or partially, through a Royal Decree. This implementation would involve making necessary adjustments or deviations to account for the specific conditions and requirements of the Faroe Islands. Essentially, it provides a framework for the potential future application of the Act in the Faroe Islands, subject to appropriate modifications as deemed necessary (Ministry of Interior and Health, 2019).

## Foreigners Seeking Employment in Greenland

Under the Greenland Parliament Act no. 27 of October 30, 1992 concerning the Regulation of Import of Foreign Workers in Greenland, priority is given to Greenlandic workers over non-Greenlandic workers for employment opportunities within the region. Employers have the responsibility to apply to the local municipality, seeking permission to hire foreign workers (Ministry of Mineral Resources and Labour, 2019).

However, Greenlandic legislation allows the utilization of foreign labor in cases where there is scarcity of qualified domestic workers. The health sector in Greenland has consistently engaged in the recruitment and employment of doctors and nurses from other Nordic countries, both for shorter and longer durations, as a prevailing practice (Preisler, 2019).

Kleemann (2023) states that the Self-Government in Greenland does not issue residence and work permits directly; however, it is consulted by the Danish Agency for International Recruitment and Integration (SIRI) to assess if the proposed terms align with local standards. The final decision regarding the granting or refusing of permits is solely with SIRI. Although Nordic nationals are not required to obtain residence and work permits to work in Greenland, employers must still obtain permission from the municipality if the job falls under the scope of Greenland Parliament Act no. 27 of October 30, 1992 on the Regulation of Import of Foreign Labour in Greenland (Kleemann, 2023).

## The Health Agreement

On 11 February, 2021, the Ministry of Immigration and Integration and the Government of Greenland reached an agreement known as the Health Agreement, facilitating easier access to work and residence permits for foreign health personnel in Greenland. This agreement allows employees in the Greenlandic health sector, such as doctors and nurses, to apply for permits under more favorable conditions than before. They may be granted permits without requiring approval from the Government of Greenland, provided they meet the requirements. Consequently, the application process for residence and work permits for this group can now be conducted in Greenland, and the target processing time has been reduced from 90 to 30 days (SIRI, 2021).

The Health Agreement expedites the issuance of work permits for doctors, nurses, and similar healthcare professionals in Greenland. In the event of securing employment within the Greenlandic Healthcare system, the Danish authorities are authorized to handle the processing of work and residence permit applications without necessitating consultation with the Greenlandic government. Eligibility requires full-time employment under the appropriate Greenlandic collective bargaining agreement, and nurses additionally need Greenlandic authorizations (SIRI, 2023). Nurses holding Danish authorization have the option to apply for Greenlandic authorization. It is also not a prerequisite to possess Danish authorization when applying for Greenlandic authorization (H. Hansen, personal communication, March 9, 2024).

## Discussion

There is a lack of research exploring the process and experiences of IENs seeking qualification as RNs in Greenland. It should be noted that the pathway for IENs seeking employment in Greenland can be complex, as they might consider obtaining a license from the STPS before seeking a separate license to work specifically as an RN. If proficiency in the Danish language is a requirement set by the STPS, it can pose challenges to IENs who have migrated to Greenland, as the official language of the country is Greenlandic (Kleemann, 2023), although Danish can be used to handle public affairs (The Prime Minister's Office, n.d.). This language barrier could have implications for effective communication with patients, particularly those who do not speak the local language properly, posing incidences of linguistic racism (Cubelo, 2023b).

In Greenland, reliance on Danish healthcare services and legislation is evident, with existing laws governing separate nurse registration. Policy reform is crucial to establish a group of diverse nursing experts under the National Board of Health responsible for IEN qualification assessment and registration policies. To address the recognition of IENs’ qualifications in Greenland, a potential solution is implementing a bridging program, demonstrated in Finland and Sweden, known for its effectiveness and satisfaction among IENs (Cubelo et al., 2023; Högstedt et al., 2021). In addition, employers should collaborate with a higher education provider in developing a bridging education program (Cubelo et al., 2023), offered in Greenlandic and Danish or English, along with organizing a skills assessment test or a national licensure examination.

Despite Greenland's self-autonomous status, the lack of specialized medical care compromises healthcare sustainability, leading to dependence on Denmark and Iceland (Gunnarsson et al., 2015). When transferring patients to Denmark, it is preferable, though not mandatory, for an IEN or local nurse to hold a Danish RN license to comply with regulations and ensure patient safety (STPS, n.d.). This highlights the need for greater cooperation and stronger regulations coordinating licensed nurses between Greenland and Denmark.

Considering the shared healthcare systems of Greenland and Denmark, improving agreements for mutual recognition of nursing qualifications is essential, particularly in emergencies requiring patient transfer to Denmark. This ensures efficient healthcare delivery while upholding professional standards and RN rights in both countries. Additionally, the Health Agreement facilitates the recruitment of Danish nurses to work in Greenland, ensuring a continued supply of healthcare professionals in the region (SIRI, 2021, 2023).

With Denmark's significant nursing shortage, the country has implemented measures to eliminate the Danish language test requirement in the application process (STPS, 2023). This change allows individuals with nursing backgrounds from outside the EU/EEA to work as nurses on a probationary basis, under the supervision of an employer, while their Danish language proficiency is evaluated. Consequently, it becomes the employer's responsibility to assess the language skills of these nurses (STPS, 2023). However, it raises the question of whether employers possess the necessary expertise to formally assess the Danish language proficiency of IENs. Additionally, considering this policy, it remains uncertain whether an IEN planning to work in Greenland during the probationary period would be required to use Danish or Greenlandic languages. The existence of Greenland's separate nursing law and setting Greenlandic as the official language introduces a potential source of confusion for IENs who intend to establish permanent residency and pursue employment in the country.

Greenland, characterized by a vast territory and a small population, often results in residents living at considerable distances from healthcare centers and regional hospitals. When individuals experience severe illness, they are transported to the nearest hospital for medical evaluation and treatment (Kleemann, 2023). Patients requiring specialized care, such as those diagnosed with cancer, receive comprehensive treatment at Queen Ingrid's Hospital in Nuuk or hospitals located in Denmark (Kleemann, 2023). In the event of critical emergencies, patients may be transferred to hospitals in Iceland (Kleemann, 2023). Given this understanding, Greenland must maximize its workforce by fully utilizing IENs who possess clinical nursing skills and educational background from non-EU/EEA countries. By doing so, the country can effectively address the issue of nursing shortages and ensure the availability of healthcare professionals. This approach highlights the potential benefits of embracing and integrating IENs into the healthcare system, ultimately contributing to the overall healthcare delivery and quality in Greenland.

## Advancing the Nursing Profession for IENs in Greenland: Strategic Political Recommendations

The reliance on Danish health services and the lack of clarity on the IEN registration process in Greenland highlight the need for policy reform. Advocating for a specialized group of diverse experts within the nursing field, particularly those IENs with extensive experience living in Greenland and a thorough understanding of the Greenlandic healthcare system under the National Board of Health, is proposed to ensure a diverse and inclusive perspective on the IEN qualification pathway. This approach will optimize regulatory processes for the unique challenges posed by the smaller and diverse cohort of IENs, fostering effective integration into the local healthcare system. The introduction of the bridging education program and a qualification assessment system would recognize Greenland's independence and ensure a pathway model for IENs to become RNs. By achieving a self-sufficient healthcare workforce, Greenland can effectively address healthcare needs within the country while still maintaining collaboration with Denmark for complex cases requiring transportation to Danish healthcare facilities.

Greenland can draw valuable insights and learn from other Nordic neighboring countries, such as Finland and Sweden, that have established pathways for recognizing the qualifications of IENs (Cubelo et al., 2023; Högstedt et al., 2021). These countries have implemented policies and programs to integrate IENs into their health systems, which can serve as a model for Greenland's policy reforms. Furthermore, it is imperative to institute an onboarding program within the social and healthcare system, addressing various challenges such as housing, kindergarten, schools meeting expectations, and employment opportunities for spouses, among other considerations (H. Hansen, personal communication, March 9, 2024).

Before migrating, IENs should have access to an official document in the English language that provides comprehensive information on various aspects relevant to Greenland. This document should cover topics such as Greenlandic healthcare legislation, specifically nursing legislation and the licensure procedure, the Greenlandic healthcare system, labor legislations in Greenland, workers’ rights protected by unions, as well as information about the local culture and available social support. To develop this essential resource, a team of specialists can be convened to create an official website that presents this information in a structured and accessible manner (see Table 2).

**Table 2.** Checklist for IENs Educated Outside the European Union/European Economic Area Seeking Employment and Recognition as Registered Nurses in Greenland.

Steps and considerations	Done	Date completed
1. *Educational background*	[ ]	
Ensure completion of a nursing program equivalent to Greenlandic standards.		
2. *Authorization process*	[ ]	
Familiarize yourself with the authorization process handled by the National Board of Health in Greenland.		
Prepare for an individual assessment considering the educational background, study duration, and relevant documentation.		
3. *Language proficiency*	[ ]	
Confirm the language requirements, acknowledging that Greenlandic is the official language but the Danish language is also accepted. Note any language certification requirements, if applicable.		
4. *Legislation understanding*	[ ]	
Gain knowledge of the legislative framework governing nursing in Greenland.		
Understand the specific regulations outlined in Parliamentary Regulation No. 7 (1995) and other related legislation.		
5. *Governmental procedures*	[ ]	
Familiarize yourself with the procedures outlined by the Greenlandic Government, especially those related to work and residence permits.		
Be aware of the Health Agreement and its implications for foreign health personnel.		
6. *Communication with authorities*	[ ]	
Establish effective communication with the National Board of Health for licensing purposes.		
Ensure a clear understanding of the roles and requirements set by the Greenlandic authorities.		
7. *Cultural competency*	[ ]	
Emphasize cultural competency and understanding of the Greenlandic environment.		
Acknowledge the importance of inclusivity and cultural diversity in the workplace.		
8. *Professional networking*	[ ]	
Seek guidance from experienced managers or professionals in the field of IEN matters.		
Connect with relevant healthcare professionals and trade unions in Greenland through official channels.		
9. *Continuous professional development*	[ ]	
Commit to continuous professional development and training.		
Stay informed about updates and changes in legislation and healthcare policies		
10. *Mental and physical wellbeing*	[ ]	
Prioritize mental and physical wellbeing, especially considering the challenges associated with migration.		
Be prepared for potential physical and mental challenges associated with work.		

*Note*. This checklist is designed to guide IENs with the key steps and considerations for seeking employment and recognition as registered nurses in Greenland. It is advisable to consult with relevant authorities and stay informed about any changes in regulations.

IENs=internationally educated nurses.

To advance the nursing profession in Greenland, several political recommendations can be implemented. Greenland can engage in the sharing of best practices by collaborating with Finland and Sweden to understand their policies and regulations to recognize IEN qualifications. This exchange of ideas can help identify effective strategies and adapt them to suit Greenland's healthcare system. It is also crucial to identify the existing competency assessment frameworks utilized in Finland and Sweden providing valuable guidance for policymakers and educators. Understanding the methodologies, criteria, and standards used to evaluate IEN competencies (Cubelo et al., 2023; Högstedt et al., 2021) can help develop a comprehensive and fair assessment process for the future development of Greenland's group of diverse nursing specialists under the National Board of Health.

The Greenlandic environment must foster inclusive and culturally diverse work environments. This involves ensuring proper staffing and equipping of facilities, along with providing institutional training and mentoring (Preziosi & Kovner, 2023). From the Nordic perspective, language support programs and cultural orientation initiatives are essential to facilitate the integration of IENs into the healthcare system (Cubelo, 2023a; Cubelo et al., 2023; Preziosi & Kovner, 2023). Greenland can study existing programs and develop language training initiatives that address language proficiency requirements (Greenlandic or Danish and English) and provide cultural orientation to help IENs adapt to the local healthcare context.

Recognizing the previous learning and experience of IENs is crucial. Greenland can explore the frameworks and procedures used by Finland and Sweden (Cubelo et al., 2023; Högstedt et al., 2021) to accurately evaluate educational credentials and professional experience. This can contribute to the development of a fair and transparent system for recognizing the qualifications and competencies of IENs based on their previous education and nursing practice. It can also be helpful to involve local nursing associations, local IEN experts, educational institutions, and healthcare employers in the development and implementation of policies to recognize IEN qualifications to ensure inclusivity and reflect local needs thus improving and protecting the public health in general.

## Limitations

To the best of the author's knowledge, this is the first scientific article discussing the pathway of IEN qualification in Greenland educated outside of the Nordic and EU/EEA countries. Further empirical research is needed on the experiences, challenges, and procedures among IENs who obtained their nurse license in Greenland that can be used for policy reforms. The economic impact of policy reform warrants further investigation to determine whether additional regulatory amendments are required for better understanding and implementation.

## Conclusion

The lack of research on the process and experiences of IENs seeking qualification as RNs in Greenland underscores the need for further investigation in this area. The complex pathway for IENs educated outside the EU/EEA region, seeking employment and RN status in Greenland, can pose challenges in attracting foreign talent to the country. Additionally, learning the Greenlandic language may present difficulties; therefore, IENs may opt to learn Danish while applying for authorization, with accessibility to language learning resources available abroad. Consequently, the language barrier creates communication challenges, especially for patients who do not speak Danish. To address these issues, policy reforms are necessary, including the establishment of a group of diverse nursing specialists under the National Board of Health responsible for assessing qualifications and registering IENs.

Implementing a bridging education program, similar to that in Finland and Sweden (Cubelo et al., 2023; Högstedt et al., 2021), could facilitate the recognition and integration of IEN qualifications. Additionally, efforts should be made to strengthen Greeland's nursing registration system which is recognized by Denmark, allowing for mutual recognition of qualifications and efficient healthcare delivery.

The use of IENs with clinical nursing skills and backgrounds outside of the EU/EEA can help alleviate the nursing shortage in Greenland and contribute to the overall healthcare system. This collaborative approach can contribute to the development of a robust and culturally competent healthcare workforce, ultimately benefiting the healthcare system and the wellbeing of the population in Greenland.

## References

[bibr1-15271544241245975] Albertsen N. Olsen T. M. Sommer T. G. Prischl A. Kallerup H. Andersen S. (2021). Who lives in care homes in Greenland? A nationwide survey of demographics, functional level, medication use and comorbidities. BMC Geriatrics, 21(1). 10.1186/s12877-021-02442-0 PMC8449891 34536989

[bibr2-15271544241245975] Bjerregaard P. Larsen C. V. L. (2018). Three lifestyle-related issues of major significance for public health among the Inuit in contemporary Greenland: A review of adverse childhood conditions, obesity, and smoking in a period of social transition. Public Health Reviews, 39, 5 10.1186/s40985-018-0085-8 PMC5901873 29692943

[bibr3-15271544241245975] Cubelo F. (2023a). Bilingual modified flipped learning in international nursing education: A discursive approach. International Journal of Nursing Sciences, 10(4), 562–567. 10.1016/j.ijnss.2023.09.006 38020832 PMC10667318

[bibr4-15271544241245975] Cubelo F. (2023b). Linguistic racism towards internationally educated nurses in Finland: Critical reflective analysis. Public Health Nursing (Boston, Mass.), 40(6), 813–816. 10.1111/phn.13233 37495551

[bibr5-15271544241245975] Cubelo F. Langari M. N. M. Jokiniemi K. Turunen H. (2023). Recognition of nursing qualification and credentialing pathway of Filipino nurses in Finland: A qualitative study. International Nursing Review. 10.1111/inr.12901 37916617

[bibr6-15271544241245975] Cubelo F. Parviainen A. Vehviläinen-Julkunen K. Palaganas E. (2024). Foreign-born nurses as COVID-19 survivors in the Nordic region: A descriptive phenomenological study. Scandinavian Journal of Caring Sciences. 10.1111/scs.13249 38404224

[bibr7-15271544241245975] Danish Patient Safety Authority (STPS) (2023). *Language test no longer required for nurses from outside the EU/EEA (Ikke længere krav om sprogprøve for sygeplejersker fra lande udenfor EU/EØS)*. Stps.dk. https://stps.dk/da/nyheder/2023/ikke-laengere-krav-om-sprogproeve-for-sygeplejersker-fra-lande-udenfor-eueoes-/?fbclid=IwAR1IggUpC7IWXhjOmS5dkkmn-oFkvHr-YSK1qCgK4qVhiNgZk1WBsR96wpU

[bibr8-15271544241245975] Danish Patient Safety Authority (STPS) (n.d.). Nurse: Application for registration. Stps.Dk. Retrieved June 28, 2023, from https://en.stps.dk/en/health-professionals-and-authorities/registration-of-healthcare-professionals/nurse-application-for-registration/

[bibr9-15271544241245975] Durnova A. Zittoun P. (2013). Discursive approach to public policy. Revue Francaise de Science Politique, 63(3), 569–577. 10.3917/rfsp.633.0569

[bibr10-15271544241245975] Exner-Pirot H. Norbye B. Butler L. (Eds.) (2018). Northern and indigenous health and health care. Saskatoon, Saskatchewan: University of Saskatchewan. openpress.usask.ca/northernhealthcare

[bibr11-15271544241245975] Greenland Self-Government (1995, October 7). *Parliamentary regulation no. 7 of 30 October 1995 on the authorisation and work of nurses (Landstingsforordning nr. 7 af 30. oktober 1995 om sygeplejerskers autorisation og virke)*. Nun.Gl. https://nalunaarutit.gl/groenlandsk-lovgivning/1995/ltf-07-1995?sc_lang=da

[bibr12-15271544241245975] Greenland Self-Government (1998, April 22). *Administrative Regulation, no. 15 of 22 April* 1998. Lovsamling. https://nalunaarutit.gl/groenlandsk-lovgivning/1998/bkg-15-1998?sc_lang=da

[bibr13-15271544241245975] Gunnarsson B. Jensen N. S. K. Garði T. I. Harðardóttir H. Stefánsdóttir L. Heimisdóttir M. (2015). Air ambulance and hospital services for critically ill and injured in Greenland, Iceland and the Faroe Islands: How can we improve? International Journal of Circumpolar Health, 74(1), 25697. 10.3402/ijch.v74.25697 26066019 PMC4463496

[bibr14-15271544241245975] Högstedt D. Engström M. Jansson I. Eriksson E. (2021). Attending a bridging program to obtain a Swedish nursing license: An interview study with internationally educated nurses. Nurse Education Today, 99, 104744. 10.1016/j.nedt.2021.104744 33549959

[bibr15-15271544241245975] Hounsgaard L. Jensen A. B. Wilche J. P. Dolmer I. (2013). The nature of nursing practice in rural and remote areas of Greenland. International Journal of Circumpolar Health, 72(1), 20964. 10.3402/ijch.v72i0.20964 PMC3753139 23984291

[bibr16-15271544241245975] Hounsgaard L. Seibæk L. (2018). Evidence-based nursing in Greenland: Pioneer spirit and long-term strategies for education and research. Nordic Journal of Nursing Research, 38(4), 175–176. 10.1177/2057158518812695

[bibr17-15271544241245975] Huang N. (2023). 10 Facts about Greenland that you might not know. Visit Greenland. https://visitgreenland.com/articles/10-facts-nellie-huang/

[bibr18-15271544241245975] International Labour Organization (2009). *Act on Greenland self-government (Act no. 473 of 12 June 2009)*. Ilo.org. https://www.ilo.org/dyn/natlex/docs/ELECTRONIC/110442/137381/F-520745313/DNK110442%20Eng.pdf

[bibr19-15271544241245975] Kleemann N. (2023). *Greenland in figures* 2023. Stat.Gl. https://stat.gl/publ/en/GF/2023/pdf/Greenland%20in%20Figures%202023.pdf

[bibr20-15271544241245975] Kočí A. Baar V. (2021). Greenland and the Faroe Islands: Denmark’s autonomous territories from postcolonial perspectives. Norsk Geografisk Tidsskrift. Norwegian Journal of Geography, 75(4), 189–202. 10.1080/00291951.2021.1951837

[bibr21-15271544241245975] Ministry of Interior and Health (1990, March 2). *BEK nr 143 af 02/03/1990, Uddannelses- og Forskningsministeriet*. Retsinformation. https://www.retsinformation.dk/eli/lta/1990/143

[bibr22-15271544241245975] Ministry of Interior and Health (2019, July 8). *LBK nr 731 af 08/07/2019, Indenrigs- og Sundhedsministeriet (Executive Order No. 731 of 8 July 2019 on authorization of healthcare professionals and health care practice)*. Retsinformation. https://www.retsinformation.dk/eli/lta/2019/731

[bibr23-15271544241245975] Ministry of Mineral Resources and Labour (2019). *Foreign workers in Greenland*. Govmin.Gl. https://govmin.gl/wp-content/uploads/2019/09/Foreign-workers-in-Greenland.pdf

[bibr24-15271544241245975] Møller S (2016). Nursing education in Greenland. Northern Review, 43, 129–133. 10.3402/ijch.v7+2i0.20964

[bibr25-15271544241245975] National Board of Health (1995). *Autorisation (Authorization)*. Landslægeembedet. https://nun.gl/emner/sundhedsprofessionelle/autorisation?sc_lang=da

[bibr26-15271544241245975] Pedersen H. B. Pedersen B. B. Biilmann M. Møller M. Lohse N. Vedsted P. Mikkelsen S. (2022). Medical evacuations in Greenland in 2018: A descriptive study. International Journal of Circumpolar Health, 81(1), 2014634–2014634. 10.1080/22423982.2021.2014634 PMC8725698 34939902

[bibr27-15271544241245975] Preisler M. (2019). *Greenland needs new jobs and foreign labour*. Nordiclabourjournal.org. http://www.nordiclabourjournal.org/nyheter/news-2019/article.2019-10-15.5136688035

[bibr28-15271544241245975] Preziosi P. Kovner C. (2023). Migrating nurses: More than addressing the U.S. nurse shortage. Policy, Politics & Nursing Practice, 24(3), 155–156. 10.1177/15271544231183857 37439018

[bibr29-15271544241245975] The Danish Agency for International Recruitment and Integration (SIRI) (2021). *New agreement regarding easier access to work and residence permits for foreign health personnel in Greenland (the Health Agreement)*. New to Denmark. https://nyidanmark.dk/en-GB/News%20Front%20Page/2021/02/Foreign-health-personnel-in-greenland

[bibr30-15271544241245975] The Danish Agency for International Recruitment and Integration (SIRI) (2023). *Work in Greenland*. New to Denmark. https://www.nyidanmark.dk/en-GB/Applying/The%20Faroe%20Islands%20and%20Greenland/Work%20in%20Greenland

[bibr31-15271544241245975] The Prime Minister’s Office (n.d.). *Greenland*. Stm.dk. Retrieved June 22, 2023, from https://english.stm.dk/the-prime-ministers-office/the-unity-of-the-realm/greenland/

